# Effectiveness of a Gamified Educational Intervention on Palliative Care Knowledge Among Nursing Students: A Single-Group Pre–Post Intervention Study

**DOI:** 10.3390/nursrep16040105

**Published:** 2026-03-25

**Authors:** Janet Vaca-Auz, Karen Jaramillo-Jácome, Melisa Chacón-Guerra, Jorge L. Anaya-González

**Affiliations:** Faculty of Health Sciences, Universidad Técnica del Norte, Ibarra 100150, Ecuador; ajvaca@utn.edu.ec (J.V.-A.); mgchacong@utn.edu.ec (M.C.-G.); jlanaya@utn.edu.ec (J.L.A.-G.)

**Keywords:** palliative care, educational intervention, nursing, gamification

## Abstract

Traditional palliative care education may limit the development of clinical competencies and attitudes required to alleviate suffering and improve quality of life. Gamification has been proposed as an alternative educational strategy in this field. **Background/Objectives**: This study aimed to assess the association between gamification-based intervention and palliative care knowledge among nursing students at a public university. **Methods**: This single-group, pre–post-intervention study was conducted in the Nursing Program of the Universidad Técnica del Norte, Ecuador, including 136 students from the accessible population. Palliative care knowledge was assessed before and after the intervention using the validated Palliative Care Quiz for Nursing (PCQN-SV). Student satisfaction and Moodle usability were assessed using a 10-item Likert-type questionnaire. The gamified educational intervention was delivered online over 60 h. Data were analyzed using descriptive statistics and Wilcoxon signed-rank tests for paired comparisons, and exploratory logistic regression analyses were conducted to evaluate contextual differences across hospitals. Statistical significance was set at α = 0.05. **Results**: The mean age was 22.9 years (SD = 1.89), and 73.5% were female. Knowledge scores increased significantly after the intervention (Wilcoxon signed-rank test, *p* < 0.001; r = 0.35). The proportion of students achieving sufficient knowledge (≥13 correct responses) increased from 27.2% (37/136) at baseline to 49.3% (67/136) post-intervention. Contextual analysis indicated variability across clinical training sites, with Lago Agrio showing higher odds of sufficient knowledge (aOR = 3.25; 95% CI [1.26–8.41]; *p* = 0.015). **Conclusions**: The gamified intervention was associated with increased palliative care knowledge among nursing students. Heterogeneity across hospitals suggests that contextual factors may influence the magnitude of change.

## 1. Introduction

Nursing education faces enormous challenges in training professionals with the skills to provide palliative care. This requires moving beyond the Tylerian curriculum model, which results in a mechanistic and technical education that is disconnected from social reality [1,2]. Current teaching strategies often prioritize memorization and procedural instruction over clinical reasoning and critical thinking. These approaches are insufficient for complex healthcare contexts [3,4,5]. The trend is to move toward the development of competencies through active and mixed methodologies that enhance meaningful learning, focused on holistic care, conceptual knowledge, communication, collaboration, and emotional preparation, with the aim of improving patient outcomes and meeting the growing need for care [4,6]. In this setting, the integration of palliative care into university nursing education is recognized as essential to prepare future professionals for the increase in chronic diseases and end-of-life needs. However, training remains heterogeneous. There is consensus on the need to establish minimum curricular content [7], as well as practical skills and ethical values that enable them to provide comprehensive, safe, and humanized care in accordance with international educational standards [8]. From this point of view, traditional teaching methods in palliative care, based on lectures and theoretical and rote learning assessments, have limitations in developing essential skills in this field, such as empathic communication, emotional management, and ethical decision-making in contexts of uncertainty [6]. In response to this, the application of innovative strategies, such as gamification, is a resource with high educational potential because it encourages active student immersion and promotes reflection and greater motivation [6,9], which contributes to strengthening learning and developing professional skills.

According to Reigada et al., gamification is a process that integrates game dynamics, mechanics, and elements into non-game contexts and has been explored as an effective method for improving knowledge, motivation, participation, interest, and learning outcomes [10,11,12]. Furthermore, the integration of gamification into MOOCs (Massive Open Online Courses) constitutes an innovative pedagogical strategy for educating and raising awareness about palliative care, allowing students to explore content in a multimodal way, face challenges, and collaborate, thus enriching their learning experience in virtual environments [10,11,13,14,15]. Among the most recent applications of gamification in the classroom are the “Bed Race, the End-of-Life Game” mission, an educational board game for teaching palliative and end-of-life care [16], and “Palliative Care Ward,” a gamified social intervention to improve learning [17,18]. Van Brakel-Van Lobenstein et al. highlight that, while implicit learning strategies promote autonomy and reflection in students, there is still insufficient evidence to support their effectiveness in developing clinical and emotional skills in real-world end-of-life care contexts [3,5]. Furthermore, Mousavinasab et al. point out that, despite the implementation of active methodologies, fragmentation in teaching persists, creating a training gap [5].

Other authors argue that introducing playful elements into sensitive educational contexts can trivialize delicate topics and detract from the emotional or ethical depth of the learning experience. From another perspective, there is debate about whether gamification can be applied to all groups and contexts [19,20,21]. In this regard, some studies suggest that the benefits of gamification depend on the participants’ prior experience and the specific design of the intervention [3,5,19,20]. In contrast to the above, several authors who have explored gamified interventions emphasize that their application has a significant impact on critical and creative thinking processes [11,20,22].

Training in palliative care is essential for nursing students, given the scientific evidence pointing to persistent deficiencies in their preparation. This situation justifies the exploration of innovative educational strategies, such as gamification through MOOCs [23]. International studies and systematic reviews show that nursing students often have insufficient levels of knowledge in palliative care. Taheri et al., through a systematic review of more than 4000 students, reported an average score of 8.94/20 points in terms of knowledge, which is considered insufficient for adequate clinical practice [24,25]. In Palestine, Alwawi et al. found that the average score was 7.42/20, confirming a lack of knowledge, although attitudes toward end-of-life care were favorable [26]. Men Cao et al. found that 50.3% of responses regarding palliative care knowledge were correct, which is moderate but still insufficient for the needs of the Chinese population [27]. This problem is exacerbated in contexts where practical training is insufficient, which affects the clinical experience acquired and the specific training that students receive [28,29]. The objective of this single-group pre–post study was to evaluate the effectiveness of a gamified educational intervention with the aim of strengthening basic knowledge about palliative care in nursing.

## 2. Materials and Methods

A single-group pre–post intervention study was conducted in the nursing program at the Technical University of the North (Ecuador) between October 2024 and February 2025 in secondary care teaching settings located in Ibarra, Otavalo, Lago Agrio, and Tena.

**Figure 1:**
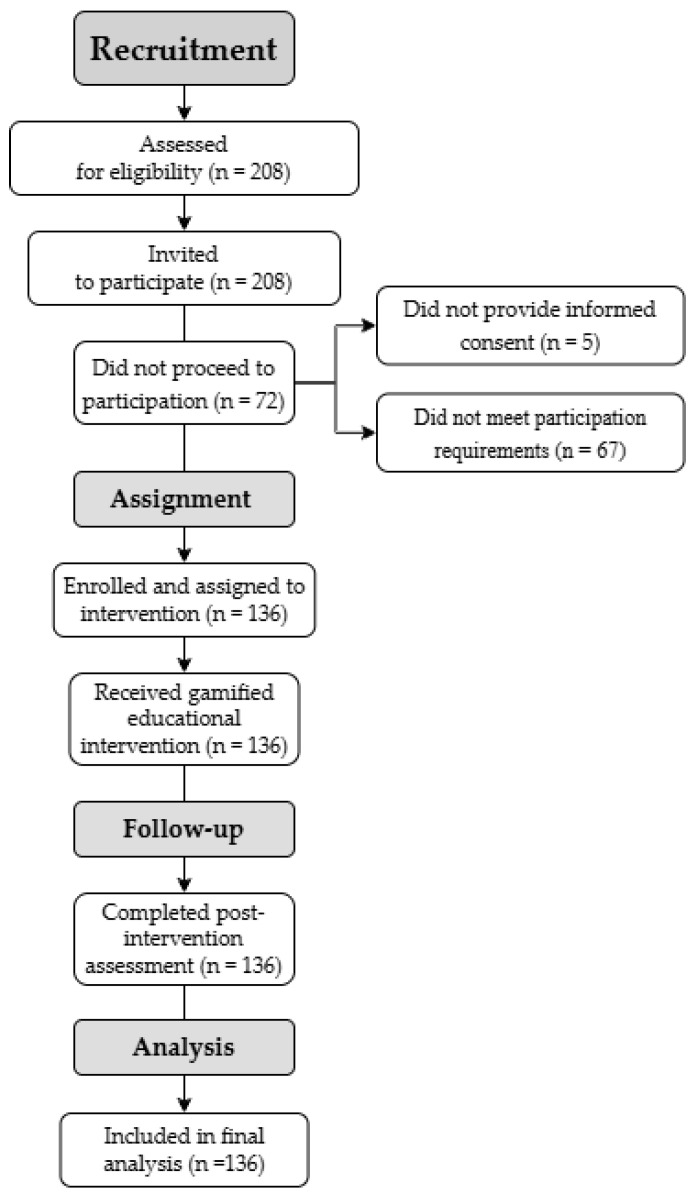
Flowchart with selection results—TREND.

During the study period, all nursing interns enrolled in the rotating internship program (N = 208) constituted the accessible population and were invited to participate. Of these, 136 students completed the intervention, and all evaluation procedures were included in the final analysis. Seventy-two students did not proceed to participate due to failure to provide informed consent (n = 5) or meet completion requirements (minimum attendance ≥ 80% and completion of assessments) (n = 67) (Figure 1).

The accessible population was distributed across two academic cohorts (May 2024–April 2025, n = 112; September 2024–August 2025, n = 96). The final analytical sample reflected a proportional distribution between cohorts (73 and 63 students, respectively), consistent with the structure of the accessible population. Baseline characteristics were comparable across cohorts.

Statistical significance was set at α = 0.05. No a priori sample size calculation was performed, as the study aimed to include the entire accessible population during the intervention period.

### 2.1. Data Collection Instruments

The pre-test was completed 15 days before the start of the educational intervention (in the pre-induction phase), and the post-test was performed 16 weeks after its completion; responses outside this defined period were excluded from the main analysis.

The demographic characteristics of the participants were collected using a structured sociodemographic questionnaire developed for this study. The instrument included the following variables: age (interval variable), sex, academic level, and area of hospital practice, selected to characterize the sample and enable subgroup analyses. The questionnaire was self-administered before the measurement of the main variables. A pilot test was conducted beforehand to ensure the comprehensibility and validity of the items.

The level of palliative care knowledge was assessed using the validated Spanish version of the Palliative Care Quiz for Nursing (PCQN-SV). This instrument consists of 20 items with a true/false/don’t know response format distributed across three dimensions: philosophy and principles of palliative care (4 items), pain and symptom management (13 items), and psychosocial aspects (3 items) [30]. Responses were coded dichotomously (correct = 1; incorrect or don’t know = 0), following the original scoring procedure. Total scores range from 0 to 20, with higher scores indicating greater knowledge [7,31,32].

At the end of the intervention, an ad hoc satisfaction survey was administered, designed based on a five-point Likert scale, aimed at assessing participants’ perceptions of the gamified methodology and the functionalities of the Moodle platform. The questionnaire included Likert-type questions, with each item presenting a statement about quality, clinical relevance, clarity of content, tutorial support, and overall satisfaction. Participants were asked to indicate their level of agreement or disagreement by selecting one of the following options: 1 = Strongly disagree, 2 = Disagree, 3 = Neither agree nor disagree, 4 = Agree, 5 = Strongly agree. In addition, the usability of the Moodle platform was evaluated, considering the clarity of the interface, the ease of access to gamified activities, and the fluidity of interactions in the virtual environment.

The instruments used showed adequate psychometric properties. Before its application, the demographic questionnaire was pilot-tested to ensure the comprehension, clarity, and relevance of the items. The PCQN-SV Scale showed adequate psychometric properties in the Ecuadorian population, with content validity indices (CVI = 0.83) and internal consistency (Cronbach’s alpha = 0.85). The process of adaptation and cross-cultural validation of instruments included expert review and pilot testing.

The satisfaction survey obtained a Cronbach’s alpha reliability coefficient (α) of 0.892 (n = 136), which demonstrates adequate internal consistency and solid coherence among the items that make up the overall satisfaction construct. Indicators related to the usability of the Moodle platform were also incorporated. These indicators strengthened content validity and allowed the instrument to reflect both pedagogical quality and technological experience.

### 2.2. Intervention

A virtual educational strategy was designed and implemented in the form of MOOCs with gamification, developed and implemented on the Moodle platform. The pedagogical design was based on meaningful learning, a patient-centered approach, and gamification as a driver of active learning aimed at achieving professional competencies. The guidelines and recommendations of the Pan American Health Organization (PAHO) [33] and the World Health Organization (WHO) were followed [34], as well as Ecuador’s technical standard for palliative care [35].

The instructional planning was structured into ten thematic modules, covering everything from the fundamentals of palliative care to ethical, humanistic, and clinical aspects. Each module incorporated multimedia resources, specialized readings, and interactive elements with a gamified approach, including informational clues, visual cues, progressive challenges, and real-time feedback to stimulate active and independent student participation. These tools facilitated meaningful knowledge building and the achievement of learning outcomes in palliative care (Table 1).

Content validation was carried out with the participation of ten specialists in palliative care, nursing, and university teaching, who were given the Delphi [36] technique in two rounds. To assess the consensus of this procedure, a scale of 0–4 was used based on the criteria of clarity, coherence, relevance, and applicability, obtaining a content validity coefficient of Aiken (V > 0.85) and a concordance index of Fleiss (k > 0.80) [37,38].

The Moodle platform, widely used in the healthcare field for its ability to store and distribute educational content, was selected to support the implementation of the proposal. Moodle was used as a central repository for teaching resources, which were designed in Dreamweaver from documents created in Word format. In addition, assessment surveys and gamified interactive resources were incorporated [18]. 

The structured instructional phase was delivered over five consecutive weeks (two modules per week). Each module consisted of six academic hours, distributed equally across tutor-led sessions, asynchronous platform-based activities, and independent study. The total workload was 60 academic hours (Table 2). Although the structured modules were completed within five weeks, the Moodle platform remained accessible throughout the rotating internship period (October 2024–February 2025) for program implementation, platform familiarization, review of supplementary materials, and content reinforcement.

**Table 1:** Structure and content of the gamified palliative care educational intervention.

Module	Content	Gamification Strategy
Module 1. Introduction to palliative care	Origins of palliative care, as well as the current state of palliative care services around the world.	Opening challenge: interactive quiz with virtual rewards (“Compassionate Caregiver Badge”) to activate prior knowledge.
Module 2. Pain: Part 1	Prevalence and proper assessment of pain, as well as the fundamental role of opioids in pain management in the context of palliative care.	Clinical role-playing: case simulations to assess and treat pain, earning points for ethical and accurate decisions.
Module 3. Pain: Part 2	Basic principles of pain management, as well as the identification and management of opioid side effects.	Therapeutic challenge: virtual board with progressive levels based on pain control and adverse effect management.
Module 4. Symptom Management: Part 1	Principles of assessment and management of common physical symptoms of dyspnea and nausea/vomiting.	Digital escape room: solve clinical cases involving respiratory and gastrointestinal symptoms to “free the patient from discomfort.”
Module 5. Symptom Management: Part 2	Principles of assessment and management of common physical symptoms of anorexia/cachexia and anxiety/depression.	Decision cards: interactive platform where students choose interventions and receive immediate feedback.
Module 6. Psychosocial and Spiritual Support	Introduction to the biopsychosocial model of care, basic principles of spiritual support and detection, and grief.	Interactive narratives: empathetic stories where the student accompanies the patient and earns points for compassionate responses.
Module 7. Ethics in Palliative Care	Review of four models of bioethical principles and how they apply to common ethical dilemmas within palliative care practice.	Gamified ethical dilemmas: selection of alternatives with scoring according to principles of autonomy, welfare, and justice.
Module 8. Communication	Introduction to basic communication skills and how physicians can respond when faced with strong emotions.	Conversation simulator: practice in virtual environments where stars are earned for empathy and communicative clarity.
Module 9. End-of-Life Care	Care for dying patients and their families using the multidimensional principles covered in modules 1–7.	Final mission: integrate prior knowledge into a complete clinical case, with an “Expert Palliative Caregiver” badge.
Module 10. Resilience and Self-Care [39,40]	Identify the increased risk of burnout among palliative care providers and highlight best practices for improving resilience and self-care.	Well-being dashboard: gamified monitoring of personal self-care with achievements for healthy practices and ethical reflection.

**Table 2:** Structural organization and workload distribution of the gamified educational intervention.

Week	Module	Tutor-Guided Hours (Synchronous)	Platform Activities (Asynchronous)	Autonomous Activities	Total Hours per Module
1	Module 1	2	2	2	6
1	Module 2	2	2	2	6
2	Module 3	2	2	2	6
2	Module 4	2	2	2	6
3	Module 5	2	2	2	6
3	Module 6	2	2	2	6
4	Module 7	2	2	2	6
4	Module 8	2	2	2	6
5	Module 9	2	2	2	6
5	Module 10	2	2	2	6
Total		20 h	20 h	20 h	60 h

### 2.3. Procedures

The study was developed and presented in accordance with the principles of the TREND (Transparent Reporting of Evaluations with Non-randomized Designs) statement, which promotes accuracy, consistency, and transparency in reports of non-randomized research [41].

The academic grading process was conducted independently of the research data analysis to avoid potential conflicts of interest.

### 2.4. Data Analysis

Continuous variables were summarized using mean and standard deviation (SD) or median and interquartile range (IQR), according to distribution. Distributional assumptions for total pre- and post-intervention scores and paired differences were evaluated using Kolmogorov–Smirnov and Shapiro–Wilk tests. As normality assumptions were not met, pre–post differences were evaluated using the Wilcoxon signed-rank test. Effect size was calculated as r = Z/√N, consistent with recommendations for non-parametric paired comparisons. Categorical variables were summarized using absolute and relative frequencies.

The primary analysis was conducted using continuous PCQN scores. For contextual exploration, sufficient knowledge (≥13 correct responses) was examined using binary logistic regression, with hospital included as a stratification variable. This approach was intended to assess differences across training settings rather than to infer causal effects of the intervention. Adjusted predicted probabilities and absolute probability differences (Δ) were estimated. Model performance was assessed using the Omnibus test of model coefficients, Nagelkerke’s R^2^, and classification accuracy.

Statistical significance was set at α = 0.05. Analyses were performed using IBM SPSS Statistics version 25.0 (IBM Corp., Armonk, NY, USA).

### 2.5. Ethical Considerations

The study was conducted in accordance with the Declaration of Helsinki and the Organic Law on Personal Data Protection of Ecuador (Official Gazette Supplement 459, 26 May 2021) [42].

The research protocol was submitted to and reviewed by the Human Research Ethics Committee (CEISH) of the Universidad Politécnica Estatal del Carchi (UPEC). On 10 December 2024, the Committee issued formal authorization and classified the study as exempt from full review under Article 40 of its internal regulations, as it did not involve invasive procedures, clinical interventions, or intentional modification of biological, psychological, or social variables. No data collection involving participants began prior to this approval.

Participation was voluntary. Eligible students were informed about the objectives, procedures, potential risks, and their right to withdraw without academic consequences. Written informed consent was obtained before inclusion.

Data were collected using coded identifiers. The database was anonymized prior to analysis, and no personal identifiers were stored. Results are presented in aggregate form to prevent individual identification.

Although the educational intervention formed part of the academic curriculum, participation in the research component and authorization for data use were optional and independent of grading.

## 3. Results

### 3.1. Pre- and Post-Intervention Analysis

The cohort included 136 nursing students. The mean age was 22.9 years (SD = 1.89). Females represented 73.5% (100/136) of the sample. The distribution by academic terms was 53.6% in Term 1 and 46.4% in Term 2.

The median total knowledge score before the intervention was 11 (IQR = 10–12). After the intervention, the median remained 11 (IQR = 11–13). Although the median remained unchanged, the interquartile range shifted upward, indicating a shift toward higher values (Table 3).

The Wilcoxon signed-rank test showed a statistically significant increase in total knowledge scores post-intervention (Z = 4.061, *p* < 0.001). The effect size was r = 0.35, indicating a moderate magnitude of change.

Before the intervention, 72.8% of students (99/136) scored below the predefined sufficiency threshold (≥13 correct responses). Following the intervention, the proportion achieving sufficient knowledge increased to 49.3% (67/136), indicating a higher concentration of students meeting the predefined competency criterion.

### 3.2. Sufficient Palliative Care Knowledge (PCQN ≥ 13) by Hospital: Contextual Variability

Stratified analysis by hospital showed no statistically significant differences in baseline sufficient knowledge (≥13 correct responses), as all adjusted odds ratios included the null value.

**Figure 2:**
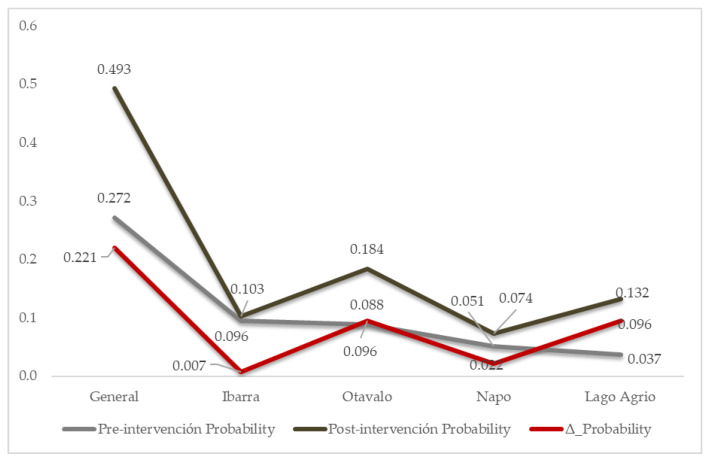
Absolute difference in adjusted probabilities of sufficient knowledge after the intervention by the hospital.

Following the intervention, Lago Agrio showed a statistically significant association with sufficient knowledge (OR = 3.25; 95% CI [1.26–8.41]; *p* = 0.015). The adjusted predicted probability increased from 0.037 to 0.132 (Δ = 0.096). Although Otavalo showed a similar absolute increase (Δ = 0.096), its confidence interval included the null value. Smaller absolute changes were observed in Ibarra (Δ = 0.007) and Napo (Δ = 0.022), indicating heterogeneity across training settings (Figure 2). These estimates reflect hospital-specific adjusted probabilities and therefore differ from the overall sample probability.

In the post-intervention analysis, the omnibus test of coefficients was significant (χ^2^ = 6.506, *p* = 0.011). Nagelkerke’s R^2^ was 0.062, indicating limited explanatory variance. Classification accuracy increased from 50.7% in the constant-only specification to 58.8% after the inclusion of the hospital.

The PCQN-SV scale assessed three dimensions with specific ranges: D1 (philosophy and principles: 0–4 points), D2 (pain and symptom control: 0–13 points), and D3 (psychosocial and spiritual aspects: 0–3 points). The pre- and post-intervention scores, together with the inferential statistics, are presented in Table 4.

Dimension D1—Philosophy and principles of palliative care: The Wilcoxon signed-rank test showed a statistically significant increase in D1 scores after the intervention (Z = 6.904, *p* < 0.001). The median score increased from 1 (pre-intervention) to 2 (post-intervention). The effect size, calculated as r, was 0.59, indicating a large magnitude of change.

Dimension D2—Pain and other symptom control: The Wilcoxon signed-rank test indicated a statistically significant increase in D2 scores after the intervention (Z = 10.110, *p* < 0.001). The median score increased from 7 to 13. The effect size (r = 0.87) indicated a very large magnitude of change.

Dimension D3—Psychosocial and spiritual aspects: The Wilcoxon signed-rank test indicated a statistically significant increase in D3 scores after the intervention (Z = 10.137, *p* < 0.001). The median score increased from 1 to 3. The effect size (r = 0.87) indicated a very large magnitude of change.

## 4. Discussion

The gamified educational intervention was associated with statistically significant improvements in all dimensions of palliative care knowledge assessed using the PCQN-SV scale. The largest effect sizes were observed in D2 (pain and symptom control) and D3 (psychosocial and spiritual aspects), both showing very large magnitudes of change (r = 0.87), followed by D1 (philosophy and principles), which showed a large effect (r = 0.59). The total score showed a moderate effect (r = 0.35). This difference may reflect the aggregation of heterogeneous domains into a composite measure.

D2 and D3 showed substantial shifts within their respective ranges. The total score integrates dispersion across items, which may attenuate the effect size. The high post-intervention medians observed in D2 and D3 suggest a possible ceiling tendency in these domains. These results are consistent with previous studies reporting performance improvements following gamified interventions in nursing students [43,44]. Given the single-group pre–post design, causal interpretation should be approached with caution.

Although D2 and D3 presented similarly large effect sizes, they represent distinct learning domains. The improvement in D2 reflects gains in learning outcomes related to pain and symptom management. The change in D3 may be linked to the immersive and reflective components incorporated into the gamified activities, which promote engagement with psychosocial and spiritual aspects of care [45,46]. This finding is relevant in light of previously reported curricular deficiencies in these humanistic areas of palliative care education [6,47].

Gamification has been incorporated into nursing education as an active learning strategy in palliative care training. Prior research indicates that structured gamified interventions improve knowledge and attitudes toward end-of-life care [6,45,48]. The present results align with this evidence.

Ausubel’s theory of meaningful learning provides a conceptual framework for interpreting these findings. The theory emphasizes the integration of new information into existing cognitive structures through contextual linkage. The interactive design of the intervention, including guided activities and feedback mechanisms, may have supported this process [49].

Our findings are comparable to those reported by Nasirzade et al. [50], where a serious game significantly increased palliative care knowledge (*p* < 0.001). That study included a randomized control group, which strengthens internal validity compared to the present design. Similarly, a structured educational module for third-year nursing students in the United Kingdom demonstrated significant improvements in PCQN scores [44], especially in opioid use and adjuvant therapies. However, persistent gaps were identified in understanding adverse effects and ethical issues related to placebo use [45].

This study has limitations inherent to single-group pre–post designs without a control group. Threats to internal validity, including maturation, history effects, or regression to the mean, cannot be excluded, particularly in D3, where baseline scores were lower. Heterogeneity across hospitals should also be considered. A statistically significant association was observed only in Lago Agrio (OR = 3.25; 95% CI [1.26–8.41]; *p* = 0.015), suggesting contextual heterogeneity not fully accounted for. Although adjustments were made for academic cohort and hospital, additional factors such as intrinsic motivation or prior informal exposure to palliative care were not measured.

The contextual differences observed across centers suggest that institutional and pedagogical conditions may influence learning outcomes. Variations in faculty experience with active methodologies, technological infrastructure, and levels of student engagement may contribute to this heterogeneity [51,52,53].

Despite these limitations, the findings support the potential role of structured gamified strategies in addressing training gaps in palliative care, particularly in symptom management and psychosocial domains. Further studies should use randomized controlled designs with active comparison groups. Knowledge retention for 3 and 6 months ought to be assessed. Mediating variables such as motivation and self-efficacy warrant formal evaluation. Integration into curricula should consider the institutional context to optimize implementation.

While causal inference is limited, the consistency of findings across dimensions and the magnitude of effect sizes support the educational relevance of the intervention.

Limitations: The single-group pre–post design without a control group restricts causal inference. The research was conducted at a single public university in Ecuador and over a limited evaluation period, which may affect generalizability to other academic settings.

Future directions: Subsequent studies should incorporate randomized or multicenter longitudinal designs and directly compare gamified, blended, and traditional instructional approaches to determine relative effectiveness and strengthen external validity.

## 5. Conclusions

The gamified educational strategy was associated with improvements in palliative care knowledge among nursing students. The largest changes occurred in symptom management and the psychosocial domain. The use of the PCQN-SV allowed standardized measurement and comparison with prior research.

The findings support the incorporation of structured game-based components within undergraduate nursing programs as complementary instructional approaches.

Future implementation should account for institutional context and faculty preparedness. Further controlled studies are required to confirm these findings and evaluate their sustainability over time.

## Figures and Tables

**Table 3:** Descriptive statistics of palliative care knowledge scores before and after the educational intervention (n = 136).

Statistic	Pre-Intervention	Post-Intervention
Median (IQR)	11 (10–12)	11 (11–13)
Minimum	7	6
Maximum	13	18

Note: Pre–post comparisons performed using the Wilcoxon signed-rank test (Z = 4.061; *p* < 0.001). Effect size r = 0.35.

**Table 4:** Pre- and post-intervention changes in palliative care knowledge scores by dimension.

Dimensions	Pre-Intervention (Median)	Post-Intervention (Median)	Z	*p*-Value	r
D1	1	2	6.904	<0.001	0.59
D2	7	13	10.110	<0.001	0.87
D3	1	3	10.137	<0.001	0.87

Note: Z = standardized Wilcoxon signed-rank statistic. Effect size calculated as r = Z/√N. All results are statistically significant (*p* < 0.001).

## Data Availability

The data supporting the findings of this study are not publicly available due to ethical and privacy restrictions related to the use of educational records of participating students. The aggregated results necessary to interpret the study are included in the article. Additional information may be requested from the corresponding author, subject to institutional and ethical approval.

## Public Involvement Statement

No public involvement in any aspect of this research. The study was conducted exclusively with undergraduate nursing students within an academic educational context. Patients, caregivers, or healthcare consumers were not involved in the design, conduct, reporting, or dissemination of this research.

## Guidelines and Standards Statement

This manuscript was drafted in accordance with the TREND (Transparent Reporting of Evaluations with Nonrandomized Designs) statement, which provides guidance for the transparent reporting of non-randomized intervention studies.

## Abbreviations

CEISH	Human Research Ethics Committee
CI	Confidence Interval
IQR	Interquartile Range
OR	Odds Ratio
PAHO	Pan American Health Organization
PCQN	Palliative Care Quiz for Nursing
SD	Standard Deviation
UPEC	Universidad Politécnica Estatal del Carchi
UTN	Universidad Técnica del Norte.
WHO	World Health Organization
Δ	Absolute Probability Difference
